# Supplementary material for: Association analysis of MTHFR (rs1801133 and rs1801131) gene polymorphism towards the development of type 2 diabetes mellitus in Dali area population from Yunnan Province, China
Source: PeerJ. 2024 Oct 24;12:e18334. doi: 10.7717/peerj.18334 (PMC11512809; doi:10.7717/peerj.18334)
Supplement: Table S1 [file peerj-12-18334-s002.docx]

Table S1 Hardy–Weinberg equilibrium of MTHFR C677T and A1298C in the control group.

| Group | Gene | Genotype | Actual frequency | Theoretical frequency | *P*-value |
| --- | --- | --- | --- | --- | --- |
| Control  (n = 272) | MTHFR C677T | CC | 110 | 116 | 0.518 |
|  |  | CT | 135 | 123 |  |
|  |  | TT | 27 | 33 |  |
|  | MTHFR  A1298C | AA | 180 | 184 | 0.293 |
|  |  | AC | 88 | 79 |  |
|  |  | CC | 4 | 9 |  |

Notes: Chi-square test for deviation from the Hardy-Weinberg equilibrium (a value of *P* < 0.05 was regarded as a deviation from the Hardy–Weinberg equilibrium).
