# Supplementary material for: Association analysis of MTHFR (rs1801133 and rs1801131) gene polymorphism towards the development of type 2 diabetes mellitus in Dali area population from Yunnan Province, China
Source: PeerJ. 2024 Oct 24;12:e18334. doi: 10.7717/peerj.18334 (PMC11512809; doi:10.7717/peerj.18334)
Supplement: Table S2 [file peerj-12-18334-s003.docx]

**Table S2 Logistic regression analysis of the effect of MTHFR C677T and A1298C gene polymorphisms in patients with T2DM.**

|  | B | S.E. | Wald | *P* | OR | 95% CI |
| --- | --- | --- | --- | --- | --- | --- |
| TT |  |  | 5.147 | 0.076 |  |  |
| CC | -0.606 | 0.269 | 5.059 | 0.024^*^ | 0.546 | 0.322–0.925 |
| CT | -0.418 | 0.259 | 2.603 | 0.107 | 0.659 | 0.397–1.094 |
| CC |  |  | 5.673 | 0.059 |  |  |
| AA | -1.220 | 0.564 | 4.674 | 0.031^*^ | 0.295 | 0.098–0.892 |
| AC | -1.010 | 0.571 | 3.128 | 0.077 | 0.364 | 0.119–1.115 |

Notes: ^*^, *P* < 0.05; ^**^, *P* < 0.01; ^***^, *P* < 0.001
