# Supplementary material for: Association analysis of MTHFR (rs1801133 and rs1801131) gene polymorphism towards the development of type 2 diabetes mellitus in Dali area population from Yunnan Province, China
Source: PeerJ. 2024 Oct 24;12:e18334. doi: 10.7717/peerj.18334 (PMC11512809; doi:10.7717/peerj.18334)
Supplement: Table S3 [file peerj-12-18334-s004.docx]

**Table S3 Comparison of Hcy level in T2DM patients who take metformin or not.**

| Variables | Not taking metformin  (*n* = 199) | Taking metformin  (*n* = 246) | *P*-value |
| --- | --- | --- | --- |
| Hcy (nmol/mL) | 7.49 ± 2.91 | 8.69 ± 3.61 | 0.072 |
