# Supplementary material for: Association analysis of MTHFR (rs1801133 and rs1801131) gene polymorphism towards the development of type 2 diabetes mellitus in Dali area population from Yunnan Province, China
Source: PeerJ. 2024 Oct 24;12:e18334. doi: 10.7717/peerj.18334 (PMC11512809; doi:10.7717/peerj.18334)
Supplement: Table S4 [file peerj-12-18334-s005.docx]

**Table S4 Comparison of Hcy level in T2DM patients who take fibric acid derivatives dyslipidemia drugs or not.**

| Variables | Not taking fibric acid derivatives drugs  (*n* = 393) | Taking fibric acid derivatives drugs  (*n* = 52) | *P*-value |
| --- | --- | --- | --- |
| Hcy (nmol/mL) | 8.01 ± 3.26 | 9.40 ± 3.97 | 0.176 |
