# Supplementary material for: Association analysis of MTHFR (rs1801133 and rs1801131) gene polymorphism towards the development of type 2 diabetes mellitus in Dali area population from Yunnan Province, China
Source: PeerJ. 2024 Oct 24;12:e18334. doi: 10.7717/peerj.18334 (PMC11512809; doi:10.7717/peerj.18334)
Supplement: Table S5 [file peerj-12-18334-s006.docx]

**Table S5 Logistic regression analysis of the effect of MTHFR C677T and A1298C gene polymorphisms in patients with T2DM and CVD.**

|  | B | S.E. | Wald | *P* | OR | 95% CI |
| --- | --- | --- | --- | --- | --- | --- |
| TT |  |  | 0.561 | 0.755 |  |  |
| CC | -0.201 | 0.329 | 0.373 | 0.541 | 0.818 | 0.430–1.558 |
| CT | -0.047 | 0.312 | 0.023 | 0.880 | 0.954 | 0.517–1.760 |
| CC |  |  | 0.480 | 0.787 |  |  |
| AA | -0.276 | 0.555 | 0.247 | 0.619 | 0.759 | 0.256–2.253 |
| AC | -0.370 | 0.570 | 0.421 | 0.516 | 0.691 | 0.226–2.110 |
